# Safety of probiotics

**DOI:** 10.1186/2045-7022-1-S1-S9

**Published:** 2011-08-12

**Authors:** Mikael Kuitunen

**Affiliations:** 1 Helsinki University Central Hospital, Skin- and Allergy Hospital, Hus, Finland

## 

Probiotics are used by millions of people for their documented or anticipated health benefits. They usu-ally belong to the genera lactobacilli and bifidobacteria and are part of the normal flora. They have been studied in a wide variety of diseases and in the prevention of inflammatory and infectious dis-eases. The safety of probiotics has been approached by evaluating retrospective reviews, prospective studies and case reports. Different probiotic bacteria have shown safe use in critically ill children, patients with Clostridium diffi-cile diarrhoea, Crohn’s disease, children attending day care centers, patients with rotavirus diarrhoea, with Helicobacter pylori infection, with irritable bowel syndrome, with HIV infection–associated diar-rhoea, with necrotizing enterocolitis, in intensive care units. Lactobacillus rhamnosus GG (LGG) is the most studied probiotic with most thorough information on safety aspects. Lactobacilli have traditionally been consumed in fermented dairy foods in many countries and have during the last 20 years been introduced into dairy products. A possible impact of LGG on occurrence of bloodstream infections by lactobacilli was evaluated. In 1990 LGG was introduced in dairy products in Finland, after a slow start a sharp rise to 6L/person/year in 1999 emerged. Since 1990 lactobacillus isolates from blood culture specimens have been performed at Helsinki University Central Hospital laboratory and a national surveillance of all bloodstream infections. Of isolates originally reported to be Lactobacillus species, the percentage among all blood cultures was 0.021 and percentage among positive blood cultures was 0.19 and in the national surveillance register 0.24. During this 10 year in-terval no increase in these figures could be observed, despite a strong increase in consumption. Lactobacillus infections are rare despite abundancy in gut normal flora and widespread use. Prudence is needed when used for immunocompromised patients, premature infants, short bowel syndrome. The benefits seem to outweigh the potential for septic infections.

